# Proposed Framework for the Evaluation of Standalone Corpora Processing Systems: An Application to Arabic Corpora

**DOI:** 10.1155/2014/602745

**Published:** 2014-12-31

**Authors:** Abdulmohsen Al-Thubaity, Hend Al-Khalifa, Reem Alqifari, Manal Almazrua

**Affiliations:** ^1^King AbdulAziz City for Science and Technology, Riyadh 11442, Saudi Arabia; ^2^King Saud University, Riyadh 12372, Saudi Arabia

## Abstract

Despite the accessibility of numerous online corpora, students and researchers engaged in the fields of Natural Language Processing (NLP), corpus linguistics, and language learning and teaching may encounter situations in which they need to develop their own corpora. Several commercial and free standalone corpora processing systems are available to process such corpora. In this study, we first propose a framework for the evaluation of standalone corpora processing systems and then use it to evaluate seven freely available systems. The proposed framework considers the usability, functionality, and performance of the evaluated systems while taking into consideration their suitability for Arabic corpora. While the results show that most of the evaluated systems exhibited comparable usability scores, the scores for functionality and performance were substantially different with respect to support for the Arabic language and *N*-grams profile generation. The results of our evaluation will help potential users of the evaluated systems to choose the system that best meets their needs. More importantly, the results will help the developers of the evaluated systems to enhance their systems and developers of new corpora processing systems by providing them with a reference framework.

## 1. Introduction

Because of the growing interest in using corpora for linguistics research, language learning and teaching, and Natural Language Processing (NLP) [1], a vast number of corpora are now available in different languages [2]. Considerable quantities of these corpora are freely available either to explore using specially designed tools via the Internet or to download as plain text files. Despite such resources, however, corpus linguists and NLP practitioners sometimes need to develop their own corpora to investigate or model special cases of language use or varieties that are not provided by available corpora.

A standalone corpus processing system is required when analyzing corpora in text formats. Specifically, this comprises a set of integrated tools to explore and investigate corpus content at different levels and to reveal language patterns in the corpus. Fortunately, many standalone corpus processing systems are currently available for use in a variety of research activities ranging from contrastive studies [3] to language teaching [4].

Although a few studies have evaluated some of the current commercially and freely available standalone corpus processing systems from various points of view, for example, Hardie [5], they do not provide a holistic framework that can be used as a reference in evaluation. This paper attempts to bridge this gap by providing a framework for evaluating standalone corpus processing systems in three dimensions, usability, functionality, and performance, while taking into consideration their suitability for Arabic corpora. The availability of such a framework will help developers to enhance and improve their systems, and also help the users of such systems choose the system that best fits their needs.

We applied our proposed evaluation framework to seven freely available corpus processing systems: aConCorde V0.4.3 (http://www.andy-roberts.net/coding/aconcorde), AntConc V3.2.4w (http://www.antlab.sci.waseda.ac.jp/software.html), Key Word in Context (KWIC) V5.0 (http://www.chs.nihon-u.ac.jp/eng_dpt/tukamoto/kwic4_e.html), Khawas V3.0 (http://sourceforge.net/projects/kacst-acptool/), Simple Concordance Program (SCP) V4.0.9 (http://www.textworld.com/), TextSTAT V2.9 (http://neon.niederlandistik.fu-berlin.de/en/textstat/), and TextStat V3.0 (http://sourceforge.net/projects/textstat/).

This paper is organized as follows.  Section 2 illustrates a brief review of some of the previous work on corpus processing system evaluation followed by a description of our proposed evaluation framework in Section 3. The results of the application of the proposed framework on the seven corpus processing systems are described in Section 4.  Section 5 comprises a discussion of the evaluation results, and our conclusions are summarized in Section 6.

## 2. Related Work

Numerous studies highlight the importance of corpora in language teaching and learning [6–8]. The expected benefits of such corpora can only be realized if there is a tool that can search and explore them efficiently. A number of corpus processing systems are either commercially or freely available. Generally speaking, these systems vary in their ease of use (usability), what they can do (functionality), and how fast and how much data they can handle (performance). To date, few evaluation studies have been conducted to assess corpus processing systems from different points of view (c.f., [4, 9–12]). The evaluated systems in these previous studies include commercially available systems such as WordSmith, free web-based systems such as Web Concordancer, and free standalone systems such as TextSTAT. Some of the evaluated systems are now out of date, either because newer versions have been released, such as AntConc 1.3, which is now in version 3.2, or because they only work with older operating systems, such as Concordancer 2.0, which works with Windows 3.1.

For the aforementioned studies, functionality evaluation mainly focused on the following six main functions: corpus file types, corpus file encoding, frequency list generation, concordance, collocation, and search results. The details provided for each of these functions vary depending on the study; in most cases, it is given as a simple description, such as whether each function is available, without any additional scoring for the system's functionality with respect to each specific function or the system in general.

As for usability, the aforementioned studies paid little attention to usability evaluation compared with functionality evaluation. Aspects such as ease of navigation, user interface, and help documentation were only mentioned and described in general terms and judged subjectively. Moreover, as was the case with functionality evaluation, no rating scale or scores were given for any of the evaluated systems.

Work by Wiechmann and Fuhs [10] is the only study that considered performance evaluation using different sized corpora, ranging from 100 K words to 100 M words. Notably, the performance of all free systems degrades when the corpus size exceeds 1 M words. Work by Hardie [5] evaluated two aspects, flexibility and usability, of an ad hoc corpus analysis tool called CQPweb. However, the usability aspect basically focused on the user friendliness of the tool (as a broad term) without specifying detailed criteria to follow.

The functionality of available standalone corpus processing tools for Arabic was examined by Roberts et al. [11]. Their main finding was that these systems are not suitable for Arabic language concordance because they do not consider the writing direction of Arabic.

In general, the main focus of these prior studies was the overall functionality of the evaluated corpus processing systems. However, there are two other important aspects that any software system evaluation must consider: usability and performance. Both of these aspects were not adequately investigated in the aforementioned studies. Additionally, in the time that has passed since those studies were published, new versions of some of the corpus processing systems have been released, and entirely new systems have also been launched, for example, Al-Thubaity et al. [13]. For these reasons, therefore, a new evaluation study of corpus processing systems is necessary.

In the following section, we introduce our framework for corpus processing systems evaluation. The framework considers the usability, functionality, and performance of the evaluated systems in more detail than previous studies by giving each of the evaluation criteria dimensions a score from which we can evaluate and compare the systems in a systematic manner.

## 3. Evaluation Criteria

Generally speaking, usability, functionality, and performance are the most important factors that affect software credibility. While usability evaluation entails many methods that can be used to evaluate any software system in general, functionality and performance evaluation are more specific to the software system type. The following subsections describe the proposed evaluation criteria for standalone corpus processing systems.

### 3.1. Usability

Usability, as an element of Human-Computer Interaction (HCI), is defined as “the ease with which a user can learn to operate, prepare inputs for, and interpret outputs of a system or component” [IEEE Std.610.12-1990]. A variety of usability evaluation methods (UEM) have emerged and evolved through research and practice in the field of usability. According to a recent survey by the Usability Professionals' Association (UPA), the methods (sometimes referred to as techniques) used by practitioners to conduct usability evaluation vary from conducting expert reviews to using satisfaction surveys [14]. For the current usability evaluation of selected corpora processing systems, we employed a modified expert review checklist from Travis [15] comprising 161 heuristics. The expert review method was used because it provides an immediate analysis of the user experience of a product.

The checklist consists of nine dimensions (see Appendix A in Supplementary Materials available online at http://dx.doi.org/10.1155/2014/602745 for a full list of the criteria), as follows.

(1) Main screen (12 criteria): this dimension relates to the main interface screen of the system. For example, the criteria ask whether it is user friendly and easy to understand and makes all available options readily accessible. Another criterion asks whether it has been professionally designed.

(2) Task orientation (25 criteria): this dimension covers how tasks are performed using the tool. For example, it investigates how easy the tasks are, whether the results are clear, and whether a novice can use it without assistance.

(3) Navigation and IA (18 criteria): this dimension focuses on the structure of the tool. For instance, the criteria investigate whether the major sections of the tool appear clearly and are present in every screen, and whether the tool is logically structured.

(4) Forms and data entry (16 criteria): this dimension relates to form design. Items include determining whether the form fields are clearly explained, whether the labels and headings are clear, and whether the forms are validated before being submitted.

(5) Trust and credibility (8 criteria): this dimension covers whether the tool is authoritative and trustworthy. For example, the criteria include whether it is clear that there is a real organization supporting the tool, and whether the tool avoids advertisements.

(6) Writing and content quality (14 criteria): this dimension relates to the content of the tool. Specifically, the criteria check whether the text is concise, with no unnecessary instructions or welcome notes, whether the most important items in a list are ranked in order of importance, whether the information is organized hierarchically, from general to specific, and whether the organization is clear and logical, such as whether the windows are easy to visually scan.

(7) Layout and visual design (29 criteria): this dimension relates to the design and layout of the tool. Specifically, the criteria check whether the layout helps to focus the user's attention on what to do next and whether things that are clickable (like icons) are obvious, such as whether the functionality of any icons and other controls are clearly labeled either by text or by their graphic design. Moreover, the criteria check whether all labels are meaningful, and whether background colors, borders, and white space have been used effectively and appropriately to help users identify sets of items as discrete functional blocks. Additionally, these criteria try to determine whether the tool has a consistent and clearly recognizable look and feel that will engage users, and whether the tool is visually attractive.

(8) Search (12 criteria): this dimension concerns the search function and search results.

(9) Help, feedback, and error tolerance (27 criteria): this dimension checks whether it is easy to get assistance in the right form and at the right time, whether prompts are brief and unambiguous, and whether users often need to consult user manuals or other external information to effectively use the tool. Moreover, this dimension checks whether the tool provides appropriate feedback when needed, whether error messages contain clear instructions on what to do next, and whether the tool does a good job of preventing users from making errors.

For each checklist item, we entered a rating of −1 (does not comply with the criterion), +1 (complies with the criterion), or 0 (partially complies with the criterion). If a criterion was not relevant to the particular tool, we left the rating blank.

### 3.2. Functionality

Users have different needs based on the types of data they have and the research questions they are considering. While some users are looking for systems that provide frequency profiles or the concordance of specific words, other users look for the distributions of words or* N*-grams across corpus texts, or they may search for the most collocated words. Using the same evaluation technique as previous research, that is, expert review, combined with our own experience with the functions that each of the evaluated systems provide, we proposed 54 evaluation criteria to examine 10 dimensions of functionality, while taking into consideration the ability of these systems to handle Arabic. These dimensions (see Appendix B for a full list of the criteria) are as follows.

(1) User interface languages (3 criteria): this dimension determined whether the user interface can be used in in one language only or if it can be switched to other languages (English, Arabic, or other languages). Systems that include different language interfaces will obviously attract more users who speak and work in those different languages, thereby making it more convenient for them to use such systems.

(2) Input text format (9 criteria): corpora files usually come in plain text format (.txt extension) as a standard format that can be processed easily. However, original corpus materials can exist in a variety of other formats, such as web pages or Microsoft Word documents. The facility of being able to include different text formats other than the standard  .txt format in a corpus will save users much time and effort. The text formats included in our evaluations are  .txt,  .doc,  .docx,  .htm,  .html,  .rtf,  .xml, and system specific formats. Any other formats were considered as one “other” format.

(3) Input text encoding (6 criteria): corpora texts can be created using a variety of encoding methods. The ability to incorporate different text encoding methods can save users much time and effort. Our evaluation includes five main encoding methods: Windows-1252, UTF (8 and 16), ASCII, Cp420, and Mac Arabic. Any other encoding methods were considered as one “other” format.

(4) Text preprocessing (4 criteria): this dimension represents an optional functionality, but it is also important because it allows users to omit certain words or characters from the corpus processing results. The accessibility of such functionality can help users to interpret the data easily and focus more on the target of their investigation. Our evaluation includes the ability to remove certain lists of words (stop lists), the ability to restrict results to certain lists of words (include lists), and the ability to remove numbers, punctuation, and other special symbols. Additionally, we consider Arabic language characteristics that may require consideration in the preprocessing phase, such as diacritics, and the normalization of (أ ,آ ,إ) to (ا) and (ة) to (ه).

(5) Single word frequency lists (3 criteria): generally speaking, the frequency list of single words is the most important function of any corpus processing system because it reveals both properties of the language and the main themes of the corpus. In our evaluation, we considered the raw frequencies of the words of the entire corpus in addition to the raw frequencies of only parts of the corpus. Additionally, as the third criterion we considered the important function of relative frequency; relative frequency information is curtailed for word frequency profile comparisons between different corpora.

(6)* N*-gram frequency lists (4 criteria): an* N*-gram refers to any consecutive* N* words.* N*-gram frequency shows how those words are related in general as well as how they come together to form meaning.* N*-gram frequency is also meaningful in language modeling in statistical Natural Language Processing (NLP) applications. We considered the same criteria here that we used for the single word frequency lists. Additionally, we checked whether the evaluated system takes into consideration the direction in which Arabic is written.

(7) Frequency profile comparisons (5 criteria): the comparison of the frequency profiles of two corpora or two different parts of the same corpora is of great importance in cases when it can be used to extract the keywords of the corpora, that is, those words that distinguish one corpus from another, or to see how certain words are used in different corpora. Our evaluation included the ease of comparing the frequency profiles of individual texts, folders, or corpora. Additionally, we checked whether the system provides a different number of formulae to carry out this comparison, and also whether the system can generate keywords of the corpus based on these formulae.

(8) Concordance (8 criteria): concordance is one of the most important functions for linguists with respect to corpora, because it indicates the contexts in which words are used. Thus, concordance can reveal the meanings of words from their actual usage. We examined the availability of functionality issues related to the concordance of single words and* N*-grams, including the concordance citation, the variability of the context window, whether the window length is measured in words or characters, the ability to sort concordance results, and, finally, whether the concordance results take into account the writing direction of Arabic.

(9) Collocation (6 criteria): generally speaking, a collocation refers to a sequence of words that often come together. Collocations are of great importance to the fields of foreign language teaching and language modeling. Different statistical measures can be used to validate the strength of word collocations. Here, we take into account the ability to produce collocations of single words and* N*-grams, the possible positions of the collocated words, whether the collocation is based on frequency or statistical measures, and whether collocation extraction takes into account the writing direction of Arabic.

(10) Saving output (6 criteria): the ability to save corpus processing results can obviously save users time by not requiring them to reprocess the corpus if they would like to examine the results again. Furthermore, the saved results can often be exported to other systems that have more sophisticated analytical tools, or they can be incorporated into other NLP systems. In our evaluation, we checked for the availability of six main data saving formats, namely,  .txt,  .svc,  .htm,  .html,  .rtf, and  .xml, and if there were any other formats, they were classified as “other.”

### 3.3. Performance

All software systems have inherent limitations on how much data they can handle and the time required to process data correctly. Knowing these system limitations helps users choose the most appropriate corpora processing system to handle their corpora effectively. To evaluate the performance of the seven corpora processing tools, we measured how long each system takes (in seconds) to display results for three functionalities, namely, word frequency, 2-gram frequency, and concordance, for three different Arabic corpora which vary in structure and size: 2012 Corpus of Arabic Newspapers (2012 CAN) (http://sourceforge.net/projects/kacst-acptool/) [13], KACST Arabic Text Classification Corpus (KACST ATCC) (Requested from Authors) [16], and the King Saud University Corpus of Classical Arabic (KSUCCA) (http://ksucorpus.ksu.edu.sa/) [17]. To achieve more accurate performance evaluation results, we furthermore split the KACST ATCC into two parts, one of 4.3 million words and the other of 7.2 million words, to have a total of five corpora for comparison.  Table 1 illustrates the number of words, number of texts, and average text length for each of the five aforementioned corpora.

## 4. Results

Based on the aforementioned evaluation criteria of usability, functionality, and performance, we used a laptop computer with 2 GB RAM and a Pentium(R) Dual-Core CPU T4400 @ 2.20 GHz, running Windows 7 Microsoft Ultimate (64-bit). We also used a small part of the KACST Text Classification Corpus during preparation to confirm the basic functionality of the systems. The main results of the evaluation results are presented in the following three subsections.

### 4.1. Usability

The usability evaluations for the seven corpora processing systems are shown in Table 2. The data show that most of the systems exhibited comparable scores in most dimensions. AntConc V3.2.4 outperformed all the other systems in terms of usability, while TextStat V3.0 had the lowest performance in terms of usability. This variation in scores might be because not all heuristics were applicable to all seven systems.

**Figure 1:**
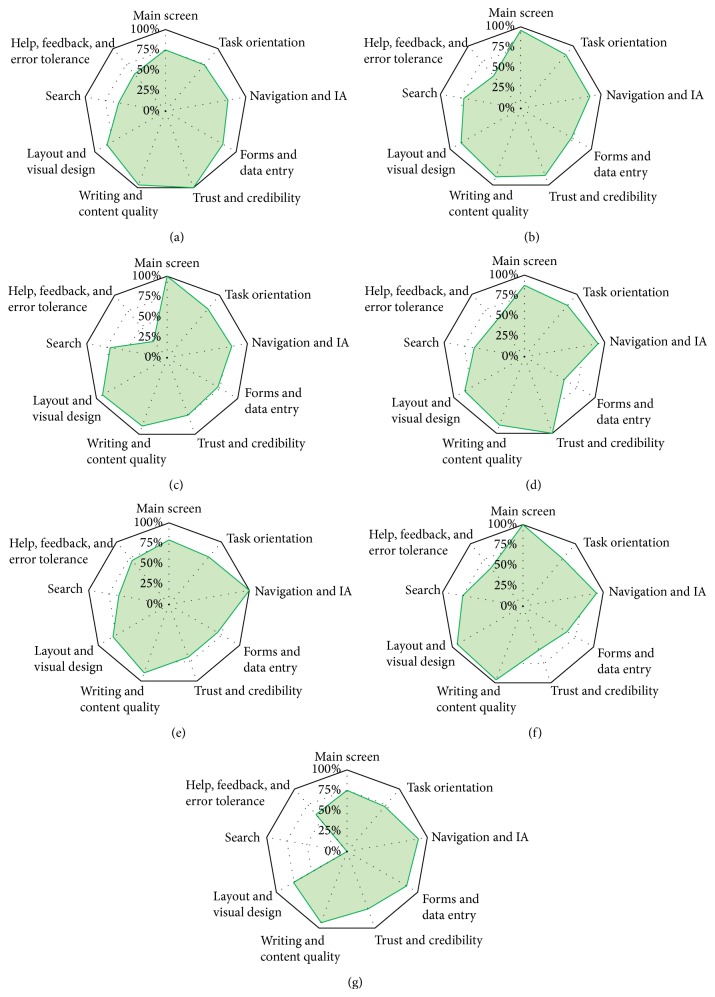
(a) Usability evaluation results for aConCorde V0.4.3. (b) Usability evaluation results for AntConc V3.2.4. (c) Usability evaluation results for KWIC V5.0. (d) Usability evaluation results for Khawas V3.0. (e) Usability evaluation results for SCP V4.0.9. (f) Usability evaluation results for TextSTAT V2.9. (g) Usability evaluation results for TextStat V3.0.

The overall score for each evaluated heuristic for the seven corpora processing systems is illustrated in Table 3. The most significant dimension that was satisfied in most systems was writing and content quality. In contrast, the help, feedback, and error tolerance dimensions fluctuated among the systems. In terms of the search factor, the 0 score achieved by TextStat 3.0 was the lowest score among the systems, indicating that such a search function does not exist in the system. However, if the TextStat 3.0 data is excluded, then the range of results for the search dimension becomes 58% to 75%; the results are similar for the help, feedback, and error tolerance dimensions. The KWIC_V5.0 system achieved the lowest score on these dimensions (26%), with AntConc3.2.4w achieving the second lowest score (52%). The results of the usability evaluation for each of the seven corpora processing systems are shown in Figures 1(a)–1(g).

### 4.2. Functionality

Each of the evaluated systems has its own strengths that distinguish its functionality from that of the other systems. However, none of the evaluated systems was able to satisfy all of the evaluation criteria; this is likely due to two factors. First, each of the systems was developed to cope with the specific processing needs of the system developers. Second, our evaluation criteria considered the ability of these systems to analyze Arabic correctly, but this was not the case for all of the corpora processing systems. The overall functionality evaluation results for each of the seven corpora processing systems are shown in Table 4. Khawas V3.0 scored the highest evaluation score, with a total score of 74%, while TextStat V3.0 scored the lowest evaluation score, with total score of 17%.

Table 5 illustrates the overall score for each of the evaluated functionality criteria. The functionality criteria that showed the best results were concordance, user interface languages, and single word frequency lists. The functionality criterion that showed the worst results, or greatest limitations, was* N*-gram frequency profile generation. Furthermore, issues related to Arabic language preprocessing and Arabic writing direction negatively affected the evaluation results. As for the user interface languages dimension, the seven corpus processing systems can be grouped into two categories, those that only use English, such as AntConc V3.2.4w, and those that use English along with other languages, such as Arabic (Khawas V3.0) and Dutch (TextSTAT V2.9).

All of the evaluated corpus processing systems accepted files with the  .txt extension as the standard input text format. The second most common file format was  .html. None of the systems accept the  .rtf format. In general, however, all of the systems were substantially varied in the number of accepted input text formats. While Khawas V3.0 accepts the  .txt format only, TextSTAT V2.9 accepts five different text formats. The results of the input text encoding dimension evaluation showed better results in cases when the system accepted at least three text encoding formats; the exception was TextStat V3.0, which accepts only one encoding format. aConCorde V0.4.3, however, outperformed all systems in this dimension. The most used text encoding format was UTF, followed by Windows-1252. This is an expected finding because both of these formats are standard text encoding formats. Also notable was the fact that all of the tested systems can read and display Arabic.

For the dimension of text preprocessing, three of the evaluated systems did not include the preprocessing of corpus texts: aConCorde V0.4.3, TextSTAT V2.9, and TextStat V3.0. As for the other four systems, the ability to remove numbers, characters, and other symbols was the most commonly satisfied criteria, while consideration of the characteristics of Arabic was only found in Khawas V3.0. Khawas V3.0 outperformed all systems in this dimension, where it satisfied all of the evaluation criteria.

The ability to analyze the raw frequency of single words for the entire corpus was one of the functionalities found in all seven of the evaluated systems. Khawas V3.0, however, was the only system that provided such ability for both single words and designated parts of the corpus. Additionally, Khawas V3.0 also provides other information, such as relative frequency, document frequency, and relative document frequency for single words.

Based on the results of our analysis, the ability to analyze* N*-Gram frequency does not seem to be of great importance in comparison to the ability to analyze single word frequency. Only two of the evaluated systems were found to provide* N*-gram frequency for the entire corpus, AntConc V3.2.4 and Khawas V3.0. Furthermore Khawas V3.0 was the only system found to satisfy all of the evaluation criteria in the* N*-gram frequency lists dimension.

Only three of the evaluated systems, AntConc V3.2.4, Khawas V3.0, and SCP V4.0.9, use basic frequency profile comparisons for keyword extraction. Of these, AntConc V3.2.4 and Khawas V3.0 use different statistical measurers to compare corpora. Khawas V3.0 is therefore the only system that satisfies all of the evaluation criteria.

Concordance is the second most common functionality available in all of the evaluated systems except for TextStat V3.0. Single word and citation concordance were the two most satisfied criteria among our evaluation criteria, while* N*-gram concordance and the consideration of Arabic language characteristics were the least satisfied criteria. Notably, three systems were found to accept the writing direction of Arabic: aConCorde V0.4.3, Khawas V3.0, and TextSTAT V2.9. Khawas V3.0, however, outperformed all other systems because it satisfied seven of the eight evaluation criteria.

Out of the seven evaluated systems, three (AntConc V3.2.4, KWIC V5.0, and Khawas V3.0) provide collocation information based on different methods. All three systems provide the collocation of single words,* N*-grams, and the position of collocated words. Furthermore, they also provide collocations based on frequency information. AntConc V3.2.4 and Khawas V3.0 equally score the highest rank in this dimension, but they are still distinguishable with respect to two functionalities; AntConc V3.2.4 provides the collocation strength based on different statistical formulae, namely, mutual information and* T*-score, while Khawas V3.0 considers the writing direction of Arabic.

All the evaluated systems allow the user to save the processed results in some way. While KWIC V5.0 allows users to copy the content, the other systems allow users to save the outputted results in a designated format. The most common designated format is plain text (.txt), followed by  .html. AntConc V3.2.4, Khawas V3.0, and SCP V4.0.9 equally outperformed the other systems in this dimension.

**Figure 2:**
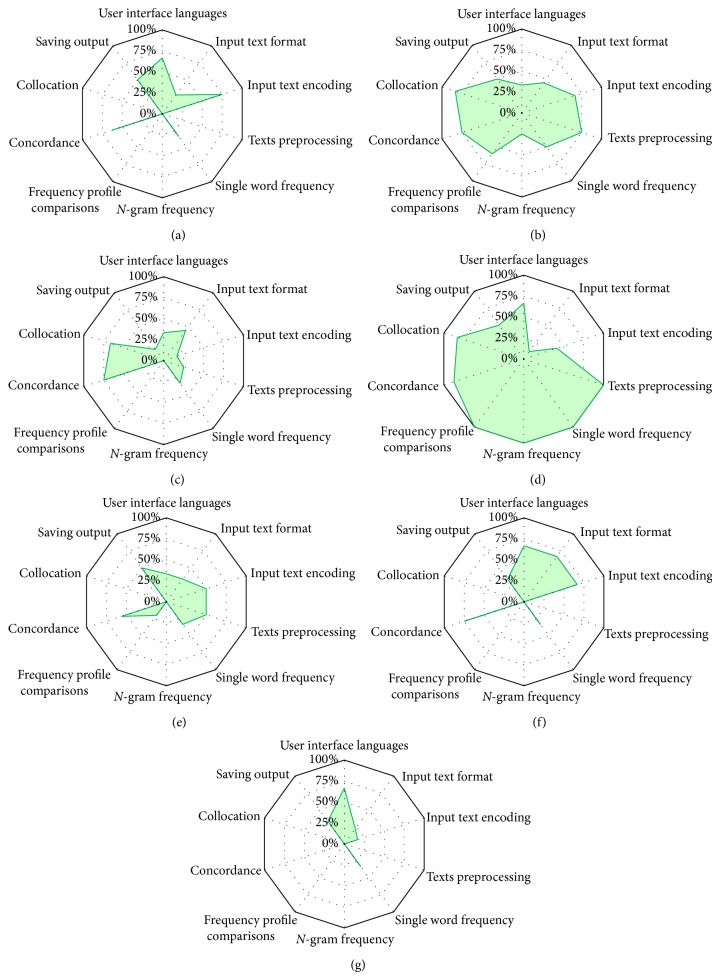
(a) Functionality evaluation results for aConCorde V0.4.3. (b) Functionality evaluation results for AntConc V3.2.4. (c) Functionality evaluation results for KWIC V5.0. (d) Functionality evaluation results for Khawas V3.0. (e) Functionality evaluation results for SCP V4.0.9. (f) Functionality evaluation results for TextSTAT V2.9. (g) Functionality evaluation results for TextStat V3.0.

The functionality evaluation results for each of the seven corpora processing systems are shown in Figures 2(a)–2(g).

### 4.3. Performance

The evaluated systems were found to vary in their performance and ability to handle the same amount of data. Performance degraded when the corpora size was increased, and in some cases the system stopped working altogether. Our performance evaluation was based on the time required by the system to generate single word frequency profiles, 2-gram frequency profiles, and concordances of the most frequent words in each of the three evaluation datasets. Two of the systems could only accept a single file as a corpus data set, aConCorde V0.4.3 and TextStat V3.0, and thus they were excluded from the current performance evaluation. Additionally, the SCP V4.0.9 system was also excluded from the performance evaluation because it truncates more than 32,000 word types.

Table 6 shows the time (in seconds) taken by the evaluated systems to display the results for the considered functionalities. For the single word frequency profiles, all systems were able to process up to 11 M-word corpora. Only two systems were able to process the KSUCCA (50 M words). Interestingly, the performance of KWIC V5.0 when processing the KSUCCA was better than its performance when processing the KACST ATCC and 7MKACST ATCC, both of which are smaller than the KSUCCA. It seems that the performance of the KWIC V5.0 system was affected by the number of texts rather than the actual number of words. In general, TextSTAT V2.9 outperformed all of the evaluated systems, while KWIC V5.0 showed the worst performance.

The performance evaluation results show that 2-gram frequency profile generation is generally a strain on the evaluated systems. Only one system (Khawas V3.0) was able to generate 2-gram frequency profiles for three datasets limited in size to 7 M words.* N*-gram frequency profile generation is a memory-consuming task that requires careful coding.

As for the dimension of concordance, all of the evaluated systems except for AntConcV3.2.4 were able to produce the concordance of the most frequent words in a corpus size up to 11 M words. This was not possible for larger corpora; all of the systems stopped working when processing the most frequent words for the KSUCCA. In general, Khawas V3.0 outperformed all of the other evaluated systems, while KWIC V5.0 was the least successful.

## 5. Discussion

The results show that the scores obtained in the usability dimension ranged between 69% and 80% with an average of 77% and standard deviation of 0.0371%. The low standard deviation indicates that the usability of the seven systems tends to be very close to one another. Additionally, they all have an acceptable user friendliness average score, that is, 77%.

However, their functionality as well as performance did not achieve acceptable results. The functionality scores ranged between 17% and 74% with an average of 39% and a standard deviation of 18.88%. This high standard deviation indicates that the functionality scores are spread over a large range of values, in turn showing that the existence of functions (and not necessarily their functionality) on some systems has helped increase their overall scores. However, the performance scores depended on the tested corpus size and criteria being evaluated (as discussed in the previous section).

Overall, the findings of our research can be summarized in the following points.

(1) The results of the usability evaluation showed comparable usability scores among all systems; however, some of the systems were found to be deficient in the dimensions of help, feedback, and error tolerance as well as search. These two dimensions are very important for the ease of use of any usable system.

(2) The results of the functionality evaluation showed a lack of ability to process the Arabic language in two main dimensions, preprocessing and collocation. Furthermore, and unrelated to the ability to process Arabic, the results showed a lack of* N*-gram frequency and collocation functionality.

(3) The importance of the performance capabilities of corpora processing systems is increased when the corpus size is increased. It is possible to overcome most of the performance difficulties encountered with larger corpora using high-end computing machines, but obviously this is not a feasible solution for all users.

(4) Only two of the evaluated corpora processing systems were able to take into consideration the characteristics of the Arabic language, aConCorde V0.4.3 and Khawas V3.0. aConCorde is limited in the amount of data it can process because it can only accept one text at a time. In contrast, Khawas V3.0 can process multiple files, up to a total size of 11 M words, and it provides different functionalities not available on aConCorde V0.4.3, or even other systems. Furthermore, Khawas V3.0 can detect the writing direction of corpus texts, which make it a reasonable choice not only for Arabic but for other languages, such as English.

## 6. Limitations and Conclusion

In the field of language resources, corpora processing systems have typically lacked any useful reference basis from which comparisons and evaluations could be made to help developers and end users when assessing such systems. In this paper we have proposed an evaluation framework for corpora processing systems based on their usability, functionality, and performance. We then applied this framework to seven corpora processing systems and compared them. Limitations of the current study result from (1) the inclusion of only seven processing systems in the comparison, (2) in-depth statistical calculations that could not be performed because the different characteristics of the evaluated processing systems made direct comparisons difficult, and (3) the application of the framework to only one language (Arabic). However, it would be interesting to apply the proposed evaluation criteria to other languages besides Arabic. One possibility is to use the proposed evaluation criteria on a well-studied language such as English and make comparisons with previous evaluation systems that have been used with English corpora.

To conclude our paper, it is worth mentioning that the proposed framework will benefit researchers and practitioners in a variety of fields (not just corpus linguistics); this includes language teachers looking for a proper corpus processing tool to produce language learning resources and software engineers who wish to consider design and functionality when building a corpus processing tool.

## Supplementary Material

Appendix A: The full list of usability evaluation criteria.
Appendix B: The full list of functionality evaluation criteria.

## Figures and Tables

**Table 1:** Number of words, number of texts, and average text length for the corpora used in the performance evaluation.

Corpus	Number of words	Number of texts	Average text length (words)
2012 CAN	2,207,469	2,910	759
4M KACST ATCC	4,356,509	5,939	734
7M KACST ATCC	7,198,767	11,719	614
KACST ATCC	11,555,276	17,658	654
KSUCCA	50,602,412	410	123,421

**Table 2:** Overall usability evaluation results for the seven corpora processing systems.

System	Usability score
aConCorde V0.4.3	78%
AntConc V3.2.4	80%
KWIC V5.0	76%
Khawas V3.0	79%
SCP V4.0.9	77%
TextSTAT V2.9	79%
TextStat V3.0	69%

Average	77%

**Table 3:** Overall score for each evaluated heuristic.

Heuristics	Average
Main screen	87%
Task orientation	78%
Navigation and IA	88%
Forms and data entry	71%
Trust and credibility	81%
Writing and content quality	92%
Layout and visual design	84%
Search	57%
Help, feedback, and error tolerance	55%

**Table 4:** Overall functionality evaluation results for the seven corpora processing systems.

System	Functionality score
aConCorde V0.4.3	30%
AntConc V3.2.4	54%
KWIC V5.0	31%
Khawas V3.0	74%
SCP V4.0.9	33%
TextSTAT V2.9	34%
TextStat V3.0	17%

Average	39%

**Table 5:** Overall score for each evaluated functionality.

Functionality	Average
User interface languages	52%
Input text format	36%
Input text encoding	48%
Texts preprocessing	36%
Single word frequency lists	45%
*N*-gram frequency lists	18%
Frequency profile comparisons	23%
Concordance	62%
Collocation	33%
Saving output	36%

**Table 6:** Time (in seconds) the evaluated systems required to display the results for the considered functionalities.

Criteria	System	2012 CAN	4MKACST ATCC	7MKACST ATCC	KACST ATCC	KSUCCA
Single word frequency	AntConcV3.2.4	32	100	170	192	SW
	KWIC V5.0	59	118	989	1010	720
	Khawas V3.0	37	61	88	117	SW
	TextSTAT V2.9	21	55	60	108	276

2-gram frequency list	AntConcV3.2.4	SW	SW	SW	SW	SW
	KWIC V5.0	NA	NA	NA	NA	NA
	Khawas V3.0	39	81	126	SW	SW
	TextSTAT V2.9	NA	NA	NA	NA	NA

Concordance of most frequent word	AntConcV3.2.4	112	312	452	SW	SW
	KWIC V5.0	106	1256	1570	1290	SW
	Khawas V3.0	24	31	56	79	SW
	TextSTAT V2.9	15	60	70	90	SW

SW: stopped working; NA: not available.
