# Supplementary material for: Proposed Framework for the Evaluation of Standalone Corpora Processing Systems: An Application to Arabic Corpora
Source: ScientificWorldJournal. 2014 Dec 31;2014:602745. doi: 10.1155/2014/602745 (PMC4294294; doi:10.1155/2014/602745)
Supplement: Supplementary file 1 — Appendix A: The full list of usability evaluation criteria. Appendix B: The full list of functionality evaluation criteria. [file 602745.f1.pdf]

Appendix A

Main Screen (12 Criteria)

1	The items on the main window are clearly focused on users' key tasks ("featuritis" has been avoided)
2	Useful content is presented on the main window or within one click of the main window
3	The main window shows good examples of real tool features
4	Buttons on the main window begin with the most important keyword (e.g. "Sun holidays" not "Holidays in the sun")
5	Navigation areas on the main window are not over-formatted and users will not mistake them for adverts
6	Navigation choices are ordered in the most logical or task-oriented manner (with the less important corporate information at the bottom)
7	All corporate information is grouped in one distinct area (e.g. "About Us")
8	By just looking at the main window, the first time user will understand where to start
9	The main window shows all the major options
10	The main window is professionally designed and will create a positive first impression
11	The design of the main window will encourage people to explore the tools
12	The main window looks like a main window; pages lower in the site will not be confused with it

Task Orientation (25 criteria)

1	The tool is free from irrelevant, unnecessary and distracting information
2	Excessive use of scripts, applets, movies, audio files, graphics and images has been avoided
3	Information is presented in a simple, natural and logical order
4	The number of screens required per task has been minimised
5	The tool requires minimal scrolling and clicking
6	The tool correctly anticipates and prompts for the user's probable next activity
7	When result are shown, users have access to the actual data (e.g. text data, corpus)
8	Users can complete common tasks quickly
9	The tool makes the user's work easier and quicker than without the system
10	The user does not need to enter the same information more than once

11	The path for any given task is a reasonable length (2-5 clicks)
12	When there are multiple steps in a task, the site displays all the steps that need to be completed and provides feedback on the user's current position in the workflow
13	Users of the tool do not need to remember information from place to place
14	The use of metaphors is easily understandable by the typical user
15	Details of the software's internal workings are not exposed to the user
16	The tool caters for users with little prior experience of the tool
17	The tool makes it easy for users to explore the tools and try out different options before committing themselves
18	A typical first-time user can do the most common tasks without assistance
19	When they use the tool again, users will remember how to carry out the key tasks
20	Action buttons (such as "Submit") are always invoked by the user, not automatically invoked by the system when the last field is completed
21	Command and action items are presented as buttons (not, for example, as hypertext links)
22	When a page presents a lot of information, the user can sort and filter the information
23	If there is an image on a button or icon, it is relevant to the task
24	The tool is robust and all the key features work (i.e. there are no javascript exceptions, CGI errors or broken links)
25	The tool supports novice and expert users by providing different levels of explanation (e.g. in help and error messages)

Navigation & IA (18 criteria)

1	There is a convenient and obvious way to move between related functions and sections and it is easy to return to the main window
2	The information that users are most likely to need is easy to navigate to from most windows
3	Navigation choices are ordered in the most logical or task-oriented manner
4	The navigation system is broad and shallow (many items on a menu) rather than deep (many menu levels)
5	The Tool structure is simple, with a clear conceptual model and no unnecessary levels
6	The major sections of the tool are available from every page (persistent navigation) and there are no dead ends
7	Tool bar are located at the top of the window, and look like clickable versions of real-world toolbar
8	There is a help that provides an overview of the tool's content
9	The help is available in every window

10	Good navigational feedback is provided (e.g. showing what you are doing in the tool)
11	Category labels accurately describe the information in the category
12	Terminology and conventions (such as link colours) are (approximately) consistent with general tool usage
13	buttons and tool bar look the same in the different sections of the tool
14	There is a visible change when the mouse points at something clickable (excluding cursor changes)
15	Navigation-only pages (such as the main window) can be viewed without scrolling
16	There are clearly marked exits on every window allowing the user to bale out of the current task without having to go through an extended dialog
17	If the tool spawns new windows, these will not confuse the user (e.g. they are dialog-box sized and can be easily closed)
18	Menu instructions, prompts and messages appear on the same place on each screen

Forms and Data Entry 16 criteria)

1	Field labels on forms clearly explain what entries are desired
2	Text boxes on forms are the right length for the expected answer
3	There is a clear distinction between “required” and “optional” fields on forms
4	Questions on forms are grouped logically, and each group has a heading
5	Fields on forms contain hints, examples or model answers to demonstrate the expected input
6	Pull-down menus, radio buttons and check boxes are used in preference to text entry fields on forms (i.e. text entry fields are not overused)
7	With data entry screens, the cursor is placed where the input is needed
8	Users can complete simple tasks by entering just essential information (with the system supplying the non-essential information by default)
9	Forms allow users to stay with a single interaction method for as long as possible (i.e. users do not need to make numerous shifts from keyboard to mouse to keyboard).
10	The user can change default values in form fields
11	Text entry fields indicate the amount and the format of data that needs to be entered
12	Forms are validated before the form is submitted
13	With data entry screens, the site carries out field-level checking and form-level checking at the appropriate time
14	The site makes it easy to correct errors (e.g. when a form is incomplete, positioning the cursor at the location where correction is required)
15	There is consistency between data entry and data display

16	Labels are close to the data entry fields (e.g. labels are right justified)

Trust and Credibility (8 criteria)

1	The tool is authoritative and trustworthy
2	The tool contains third-party support (e.g. citations, testimonials) to verify the accuracy of information.
3	It is clear that there is a real organisation behind the tool (e.g. there is a physical address or a photo of the office)
4	The company comprises acknowledged experts (look for credentials)
5	Each window is clearly branded so that the user knows he is still using the same tool
6	The tool is free of typographic errors and spelling mistakes
7	The visual design complements the brand and any offline marketing messages
8	There are real people behind the organisation and they are honest and trustworthy (look for bios)

Writing & Content Quality (14 criteria)

1	Text is concise, with no needless instructions or welcome notes
2	windows use bulleted and numbered lists in preference to narrative text
3	Lists are prefaced with a concise introduction (e.g. a word or phrase), helping users appreciate how the items are related to one another
4	The most important items in a list are placed at the top
5	Information is organised hierarchically, from the general to the specific, and the organisation is clear and logical
6	windows are quick to scan, with ample headings and sub-headings and short paragraphs
7	Each window is clearly labelled with a descriptive and useful title that makes sense as a bookmark
8	Buttons and button titles are descriptive and predictive, and there are no “Click here!” buttons
9	The tool avoids cute, clever, or cryptic headings
10	Button names match the title of destination window, so users will know when they have reached the intended window
11	Button labels and link labels start with action words
12	Headings and sub-headings are short, straightforward and descriptive
13	The words, phrases and concepts used will be familiar to the typical user
14	Acronyms and abbreviations are defined when first used

Page Layout and Visual Design (29 criteria)

1	The screen density is appropriate for the target users and their tasks
2	The layout helps focus attention on what to do next
3	On all windows, the most important information (such as features and functions) is presented on the first screenful of information ("above the fold")
4	The tool can be used without scrolling horizontally
5	Things that are clickable (like buttons) are obviously pressable
6	Items that aren't clickable do not have characteristics that suggest that they are
7	The functionality of buttons and controls is obvious from their labels or from their design
8	Clickable images include redundant text labels (i.e. there is no 'mystery meat' navigation)
9	Fonts are used consistently
10	The relationship between controls and their actions is obvious
11	Icons and graphics are standard and/or intuitive (concrete and familiar)
12	There is a clear visual "starting point" to every window
13	Each window of the tool shares a consistent layout
14	Windows of the tool are formatted for printing, or there is a printer-friendly version
15	Buttons and links show that they have been clicked
16	GUI components (like radio buttons and check boxes) are used appropriately
17	Fonts are readable
18	There is a good balance between information density and use of white space
19	The tool is pleasant to look at
20	The tool avoids extensive use of upper case text
21	The tool has a consistent, clearly recognisable look and feel that will engage users
22	Saturated blue is avoided for fine detail (e.g. text, thin lines and symbols)
23	Colour is used to structure and group items on the window
24	On content windows, line lengths are neither too short (<50 characters per line) nor too long (>100 characters per line) when viewed in a standard browser width window
25	Windows have been designed to an underlying grid, with items and widgets aligned both horizontally and vertically
26	Meaningful labels, effective background colours and appropriate use of borders and white space help users identify a set of items as a discrete functional block

27	Individual windows are free of clutter and irrelevant information
28	Standard elements (such as window titles, tool bar , privacy policy etc.) are easy to locate
29	Icons are visually and conceptually distinct yet still harmonious (clearly part of the same family)

Search (12 criteria)

1	The search results window shows the user what was searched for and it is easy to edit and resubmit the search
2	The search results window makes it clear how many results were retrieved, and the number of results per window can be configured by the user
3	If no results are returned, the system offers ideas or options for improving the query based on identifiable problems with the user's input
4	The search engine handles empty queries gracefully
5	The search engine includes templates, examples or hints on how to use it effectively
6	The search results window does not show duplicate results (either perceived duplicates or actual duplicates)
7	The search box is long enough to handle common query lengths
8	Searches cover the entire tool, not a portion of it
9	The search box and its controls are clearly labelled (multiple search boxes can be confusing)
10	The scope of the search is made explicit on the search results window and users can restrict the scope (if relevant to the task)
11	The search results window displays useful meta-information, such as the size of the document, the date that the document was created and the file type (Word, pdf etc.)
12	The search engine provides automatic spell checking and looks for plurals and synonyms

Help, Feedback and Error Tolerance (24 criteria)

1	The FAQ or on-line help provides step-by-step instructions to help users carry out the most important tasks
2	It is easy to get help in the right form and at the right time
3	Prompts are brief and unambiguous
4	The user does not need to consult user manuals or other external information to use the tool
5	The tool provides good feedback (e.g. progress indicators or messages) when needed
6	Users are given help in choosing functions
7	User confirmation is required before carrying out potentially “dangerous” actions

	(e.g. deleting something)
8	Confirmation windows are clear
9	Error messages contain clear instructions on what to do next
10	When the user needs to choose between different options (such as in a dialog box), the options are obvious
11	Error messages are written in a non-derisory tone and do not blame the user for the error
12	Windows load quickly (5 seconds or less)
13	The tool provides immediate feedback on user input or actions
14	When giving instructions, windows tell users what to do rather than what to avoid doing
15	The tool shows users how to do common tasks where appropriate (e.g. with demonstrations of the tool's functionality)
16	The tool provides context sensitive help
17	Help is clear and direct and simply expressed in plain English, free from jargon and buzzwords
18	The tool provides clear feedback when a task has been completed successfully
19	There is sufficient space between targets to prevent the user from hitting multiple or incorrect targets
20	There is a line space of at least 2 pixels between clickable items
21	The tool makes it obvious when and where an error has occurred (e.g. when a form is incomplete, highlighting the missing fields)
22	The tool uses appropriate selection methods (e.g. pull-down menus) as an alternative to typing
23	The tool does a good job of preventing the user from making errors
24	The tool ensures that work is not lost (either by the user or tool error)
25	Error messages are written in plain language with sufficient explanation of the problem
26	When relevant, the user can defer fixing errors until later in the task
27	It is easy to “undo” (or “cancel”) and “redo” actions

Appendix B

User Interface Languages (3 criteria)

1	Arabic
2	English
3	Other Languages

Input Text Format (9 criteria)

1	TXT
2	DOC
3	DOCX
4	HTML
5	HTM
6	XML
7	RTF
8	System Specific
9	Other

Input Text Encoding (6 criteria)

1	Windows-1252
2	UTF
3	ASCII
4	Cp420
5	Mac Arabic
6	Other

Text Preprocessing (4 criteria)

1	Remove numbers, punctuation and Latin characters,
2	Normalization (Diacritics, Alef and Taa Marbutah.)
3	Stop list
4	Include list

Single Words Frequency Lists (3 criteria)

1	Single word frequency for entire corpus
2	Single word frequency for part of the corpus
3	Additional single word profiles (Document frequency, Relative Frequency, …)

N-Gram Frequency Lists (4 criteria)

1	N-grams, Frequency for entire corpus
2	N-grams, Frequency for part of the corpus
3	Considering Arabic writing direction
4	Additional N-Grams profiles (Document frequency, Relative Frequency, …)

Frequency Profile Comparisons (5 criteria)

1	**across files**
2	across folders
3	across different corpora
4	different No. of comparison formula
5	Key words extraction

Concordance (8 criteria)

1	Single word
2	N-grams
3	Citation
4	Character Window type
5	Word Window type
6	Window variability
7	Results sorting
8	Considering Arabic writing direction

Collocation (6 criteria)

1	Single word
2	N-grams
3	Position
4	Collocation strength (formula , no. of formula)
5	Collocation Strength (frequency)
6	Considering Arabic writing direction

Saving Output (6 criteria)

1	TXT
2	CSV
3	HTML, HTM
4	XML
5	RTF
6	Others
